# Chlorophytes response to habitat complexity and human disturbance in the catchment of small and shallow aquatic systems

**DOI:** 10.1038/s41598-022-17093-3

**Published:** 2022-07-29

**Authors:** Sofia Celewicz, Anna Kozak, Natalia Kuczyńska-Kippen

**Affiliations:** 1 Department of Botany, Faculty of Agronomy, Horticulture and Bioengineering, Poznań University of Life Sciences, Wojska Polskiego 71 C, 60-625 Poznań, Poland; 2 Department of Water Protection, Faculty of Biology, Adam Mickiewicz University, Uniwersytetu Poznańskiego 6, 61-614 Poznań, Poland

**Keywords:** Ecology, Environmental sciences

## Abstract

Human-originated transformation in the catchment area may be reflected in the water quality and ecological state of the aquatic environment. Chlorophytes, the most common and diverse group of microalgae, may be a valuable tool for studies of small water bodies, ecosystems poorly recognized but extremely sensitive to the climate changes. Here we investigated the response of the chlorophytes to abiotic and biotic factors in different habitats and ponds’ catchments. Chlorophytes demonstrated a prevalence towards a specific type of catchment area. Field ponds supported chlorophytes typical for nutrient-rich/high-organic and shallow well-mixed waters. Forest ponds supported high chlorophyte diversity. A high importance of desmids, tolerant to light deficiency, confirms their preferences towards lower pH and lower trophic state in the forest ponds. Habitat type strongly impacted the distribution of chlorophytes. Great abundance and fertile-water species were associated with the open water, whereas aquatic plants hosted relatively low chlorophyte abundance which is a derivate of the filtrators grazing as well as the nutrient uptake and shadowing by macrophytes. Macrophyte-dominated zones created favorable conditions for some periphytic desmids and filamentous chlorophytes, species preferring lower trophic state and co-occurring with zooplankton. We assume that cosmopolitan chlorophytes can be adapted for determination of the ecological value of small water bodies, including the level of habitat heterogeneity. But chlorophytes clearly react to the level of human impact in the ponds’ catchment, both specific species and functional groups. Thus, we recommend them, particularly desmids, for water quality state assessment in ponds.

## Introduction

Chlorophytes are the most diverse taxonomical group of algae, inhabiting all types of water bodies within marine, freshwater, and terrestrial ecosystems^1^. They often dominate in freshwaters, thus playing a basic role in the functioning of many aquatic environments. Most of chlorophyte species (except desmids) prefer rather warm and fertile waters^2^ but are also considered as cosmopolitan and ubiquistic organisms. For this reason, this group of algae has rarely been applied for bioindication purposes related to water state, compared with e.g. diatoms or cyanobacteria^3,4^. Most studies concern the relationship between green algae and physicochemical parameters of water, e.g. temperature, pH, O_2,_ N and P concentrations^5^. Even though some data exists on using some taxa in monitoring freshwater trophic state e.g.^6^, there is still a lack of detailed information regarding the response of microalgal chlorophytes to broadly understood ecological conditions, particularly in case of small water bodies. Only random and detached information is available on the impact of land use in the catchments e.g.^7^ or the effect of various aquatic vegetation types on the occurrence of green algae e.g.^8,9^. However, these environmental factors may be of key importance in structuring communities of organisms. Therefore, to fill the knowledge gap with respect to the role of chlorophytes in analyses of habitat heterogeneity referring to various habitats, and in particular of the role of human-induced transformation in the catchment area, thorough studies were carried out on a large group of small water bodies.

In pond-type of water bodies, environmental variables controlling the microalgae community structure are not exactly the same as those occurring in larger water ecosystems. Due to their small area and shallow character, the life conditions in ponds may be more favourable for chlorophytes. This is connected with rapid heating of waters, highly required flow of sunlight to the bottom, better availability of nutrients and their easy transfer to the productive surface zone as well as large variety of microhabitats^10^. Moreover, great fluctuations of physical–chemical variables in small water bodies create specific but chanegeable conditions for inhabiting microalgae. It would be expected that in the studied ponds, which are typical unstable environments, Chlorococcales algae, with small cell dimensions, fast reproduction and short lifespan^2,11^, will dominate. They are primary producers and are often an important part of the diet of primary consumers in aquatic ecosystems^12,13^. Zooplankton grazing activity on phytoplankton typically depends on prey concentration, and its usual selectivity is a function of prey size. The diversity of zooplankton communities relies on a number of environmental factors. However, certain groups of zooplankton will have a different impact on the structure of a prey community. Various studies e.g.^14,15^ show that larger-sized zooplankton, filtrators, will usually exert a stronger effect on phytoplankton. Levine et al.^16^ found that macrozooplankton fed most selectively on dinoflagellates and chlorophytes rather than on cryptophytes, diatoms or cyanobacteria. Moreover, small-bodied crustaceans are known to consume cryptophytes, non-filamentous diatoms, green algae, or even colonial cyanobacteria^17^. Conversely, microzooplankton such as rotifers consumed diatoms, cyanobacteria and cryptophytes, but rather avoided green algae. The effect of zooplankton grazing on green algae is even lower when filamentous forms or colonial chlorophytes such as *Volvox* predominate^18^.

According to some authors examining ponds e.g.,^19–21^ the type of surrounding of the water body has the greatest impact on the physicochemical parameters and can reflect the level of human impact^22^. Specifically, it refers to the variation in the degree of transformation in the direct catchment area. Ponds within forested area are less exposed to anthropopressure, compared to the field ponds, which are loaded with nutrients leaching from the neighbouring arable fields (natural and artificial fertilizers). Furthermore, field ponds may undergo the effect of close vicinity of farms and rural infrastructure, which contribute greatly to the environmental degradation as well as pollution^23^ and disturb the entire functioning of a pond. Therefore, it can be expected that the structure of chlorophyte communities in field and forest ponds will vary. Additionally, ponds belong to highly critical freshwater ecosystems for maintaining a high level of wildlife diversity. Because of their generally large abundance and thus greater total area than lakes, ponds contribute to an extremely high biodiversity of both flora and fauna^24,25^. However, their poor morphological features, such as small size and depth, can result in great exposure to severe human disturbances^26,27^. Thus, deterioraion of freshwater biodiversity happens in response to habitat destruction, eutrophication and land use, while habitat heterogeneity referring to the maintenance of various microhabitats even within the small area of a pond will have a positive effect^28^.

The impact of submerged macrophyte beds on microalgae, an issue rarely studied in small water bodies, is also very important. Both diversity and abundance of macrophytes will contribute to an increase in ecological value but this is also a derivative of human impact in the catchment area. Aquatic plants create a specific mosaic of microhabitats for planktonic organisms thereby having an important structuring impact on communities of plankton^29,30^. For zooplankton, they also serve as a shelter from predators^31,32^. Macrophytes can affect algal assemblages through creating ecological niches enabling the co-existence of many species. They can also influence algae through the competition for nutrients and light^33^, by secretion of allelopathic substances which inhibit algal growth^34^ or change morphology^35^ and indirectly, by modifying some physicochemical parameters of water^36,37^.

The majority of phycological studies conducted in ponds have only concentrated on abiotic factors that influence microalgal communities. However, there is a need for comprehensive analyses that would take into account both abiotic (in ponds of different types of catchments) and biotic factors (e.g. competition with macrophytes and grazing by zooplankton) that will determine chlorophyte communities, which are often a leading group in freshwater habitats. The recognition of the habitat preferences of green algae in ponds with various microhabitats and determination of the selectivity for a certain pond type, located within different types of catchments, will allow a better understanding of the functioning of aquatic food webs.

It is a well known phenomenon that plankton reacts rapidly to ecological changes and can be therefore a good indicator of water quality due to their short lifetime and rapid rate of reproduction^38^. The results of our study will show the potential usefulness of individual species of chlorophytes as indicators in the biological monitoring of small water bodies. While individual species of algae can occur either seasonally or only locally due to, for example, the specificity of the habitat or local environmental conditions, functional groups of organisms can be used universally and can create an excellent comparative tool for the assessment of small water bodies located in different regions of the world. The species response to environmental conditions determines their functional traits. Therefore, in order to examine the algae preferences more closely, the analyses included not only the influence of environmental variables on the particular taxa in different microhabitats and ponds within various catchments, but also functional groups of microalgae^39,40^, to which these taxa have been assigned.

Even though chlorophytes are characterised by great species and morphological diversity and their frequent dominance in freshwater habitats often makes them a key element in trophic webs, there is still a lack of information on their occurrence in various habitats and particularly in small water bodies undergoing varying impact of the catchment area. Therefore, we hypothesised that chlorophytes can be used as a very valuable indicator of habitat heterogeneity (the open water area vs. macrophyte stands). Furthermore, we assumed that chlorophytes, which are often used for ecological studies (functional groups, trophic state determination in lakes), can also be implemented to assess the type of pond surroundings and specifically the level of human impact (field vs. forest ponds).

The main purpose of the study was to detect patterns in chlorophyte diversity and community structure and to relate these patterns to various habitats and various catchment types. The research aims included: (1) finding out the best drivers of chlorophyte species diversity (2) eliciting the best predictors for the distribution of chlorophyte species in ponds in two varying types of surroundings (field vs. forest); (3) extracting specific habitat preferences of certain chlorophyte species (water vs. macrophytes); (4) application of functional groups of chlorophytes for ecological state assessment.

This research will contribute to a better understanding of the functioning of poorly studied small water bodies, thanks to the analysis of the community structure of green algae—the most numerous, often dominant and diverse group of phytoplankton in freshwater environments. It is particularly essential in the context of climate warming, as green algae prefer higher water temperature^41^. Therefore, it should be expected that their share and importance, especially in fast-warming small water bodies, will increase, and not only in the seasonal aspect—in the summer—in the case of the temperate climate zone. Ponds are the aquatic ecosystems most vulnerable to climate change, but also to human impact referring to agriculture and urban development. Therefore, interdisciplinary research conducted on this type of water body is extremely important and valuable, although, unfortunately, less frequently conducted than in larger systems.

## Results

Chlorophytes constituted a great part (233 taxa in total and on average about 33% per sample; range: 6–75% of the total algae species composition) of the phytoplankton species diversity in the investigated ponds.

Species such as *Raphidocelis danubiana, Tetraëdron minimum, Oocystis lacustris* had the highest abundance in the field ponds. They were also characterised by high frequency. In forest ponds species such as e.g. *Kirchneriella cornuta, Monoraphidium circinale* and *Dictyosphaerium ehrenbergianum* were abundant, although their frequency was lower (see Appendix S1). All of these species are common in freshwater bodies in the temperate climate zone. Most of them are small single-celled chlorophytes from the order Sphaeropleaes, except those forming colonies (*Oocystis lacustris* and *Dictyosphaerium ehrenbergianum*), which belong to the order Chlorellales.

**Figure 1:**
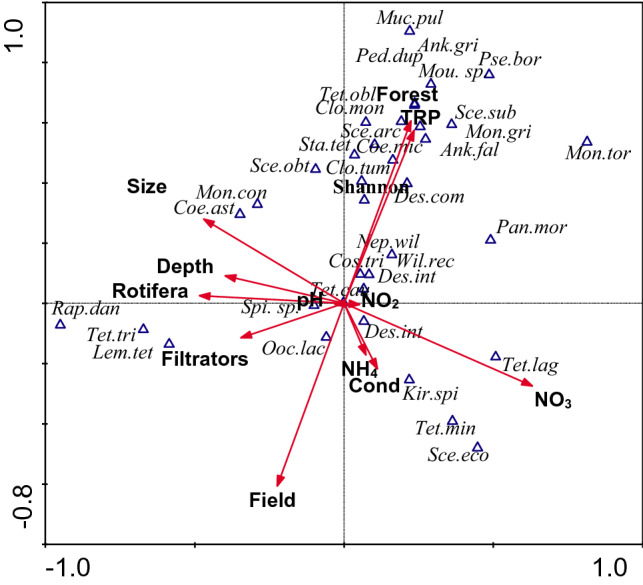
CCA diagram of the distribution of chlorophyte dominating species and diversity (Shannon—Shannon–Wiener Diversity Index) in relation to environmental factors in field and forest ponds. Dominating taxa/Abbreviation: Ank.arc—*Ankistrodesmus arcuatus*; Ank.fal—*Ankistrodesmus falcatus*; Clo.mon—*Closterium moniliferum*; Clo.tum—*Closterium tumidulum*; Coe.ast—*Coelastrum astroideum*; Coe.mic—*Coelastrum microporum*; Cos.tri—*Cosmarium trilobulatum*; Des.arm—*Desmodesmus armatus*; Des.com—*Desmodesmus communis*; Des.int—*Desmodesmus intermedius*; Kir.spi—*Kirchneriella irregularis* var. *spiralis*; Lem.tet—*Lemmermannia tetrapedia*; Mon.con—*Monoraphidium contortum*; Mon.gri—*Monoraphidium griffithii*;Mon.tor—*Monoraphidium tortile*; Mou.sp.—M*ougeotia* sp.; Muc.pul—*Mucidosphaerium pulchellum*; Nep.wil—*Nephrochlamys willeana*; Ooc.lac—*Oocystis lacustris*; Pan.mor—*Pandorina morum*; Ped.dup—*Pediastrum duplex*; Pse.bor—*Pseudopediastrum boryanum*; Rap.dan—*Raphidocelis danubiana*; Sce.arc—*Scenedesmus arcuatus* var. *gracilis*; Sce.eco—*Scenedesmus ecornis*; Sce.obt—*Scenedesmus obtusus*; Sce.sub—*Scenedesmus subspicatus*; Spi.sp.—*Spirogyra* sp.; Sta.tet—*Stauridium tetras*; Tet.cau—*Tetraedron caudatum*; Tet.min—*Tetraedron minimum*; Tet.obl—*Tetradesmus obliquus*; Tet.lag—*Tetradesmus lagerheimii*; Tet.tri—*Tetraedron triangulare*; Wil.rec -*Willea rectangularis*.

**Figure 2:**
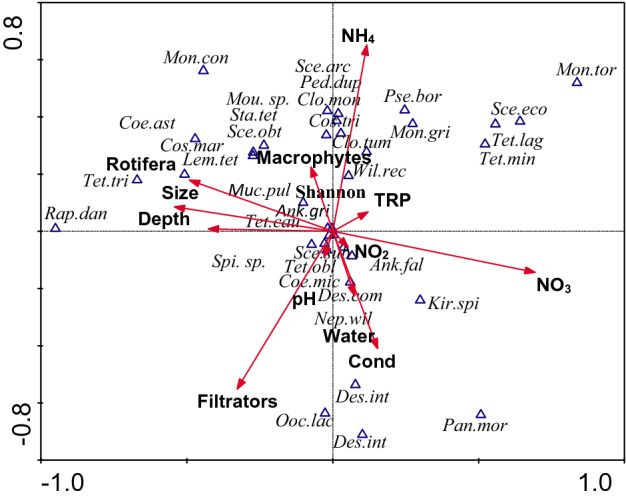
CCA diagram of the distribution of chlorophyte dominating species and diversity in relation to environmental factors in two types of pond habitats (Water—open water and Macrophytes—macrophyte-dominated zones). Abbreviation: Cos.mar—*Cosmarium margaritatum*; other see Fig. 1.

From among chlorophyte taxa, 36 were taken into CCA analyses (Figs. 1, 2).

### Relationships between chlorophytes and environmental variables in different pond types (field vs. forest)

The mean values of water temperature, pH, conductivity were significantly higher in field ponds, while the pond size and the level of water saturation were lower here compared with forest ponds (Table 1). In contrast to the forest ponds, the field ponds were also characterised by slightly higher concentrations of TRP and mean values of chlorophytes, Rotifera and filtrator abundance. Other examined parameters did not reveal any variation between these two pond types.

**Table 1:** Limnological parameters (*Temp* water temperature, *pH* water reactivity, *Cond* conductivity, *O*_*2*_ water saturation, *TRP* total reactive phosphorus, *DIN* dissolved inorganic nitrogen, *NH*_*4*_ ammonium, *NO*_*3*_ nitrates, *NO*_*2*_ nitrites), number of chlorophytes taxa (N taxa) and individuals (No. ind.), phytoplankton diversity (Shannon—Shannon–Wiener Diversity Index) and zooplankton (Rotifera, Filtrators) abundance of different pond types (field vs. forest).

Type of pond		Field				Forest				Mann–Whitney test	
Parameter	Unit	n samples	$\overline{x }$	Range	SD	n samples	$\overline{x }$	Range	SD	Z	p
Size	ha	81	0	0.002–2	0	47	1	0.007–4	1	− 2.89	< 0.01
Depth	m	81	1	0.1–7	1	47	1	0.15–4	1	–	–
Temp	^o^C	81	24	10–35	4	47	22	14.1–28	4	3.42	< 0.001
pH		80	8	6.7–11	1	47	8	6.3–10	1	1.99	< 0.05
Cond	µS cm^−1^	81	970	108.7–2078	450	47	491	26–1085	202	6.12	< 0.001
O_2_	%	81	80	3–178	44	47	104	22–259	55	− 2.08	< 0.05
TRP	µg l^−1^	81	294	0.0088–2181	487	47	82	1–590	127	–	–
DIN	mg l^−1^	81	2	0.253–9	1	47	5	0.743–160	23	–	–
NH_4_	mg l^−1^	81	1	0–5	1	47	1	0.3034–6	1	–	–
NO_3_	mg l^−1^	81	1	0–8	1	47	1	0.0266–4	1	–	–
NO_2_	mg l^−1^	81	0	0	0	47	0	0–1	0	–	–
N taxa		81	20	1–53	12	47	21	2–46	11	–	–
No. ind	ind. ml^−1^	81	3,978,155	0.006–156,400,005	18,108,293	47	1,347,681	0.01–14,489,000	3,068,527	–	–
Shannon		81	2	0–3	1	47	2	0–3	1	–	–
Rotifera	ind l^−1^	81	4368	5.6667–42,795	8786	47	2549	7.5–14,036	3118	–	–
Filtrators	ind l^−1^	80	476	0–4063	924	47	275	0–2715	509	–	–

The results of Mann–Whitney test are given.

The results of CCA analyses showed that the type of catchment area had a significant impact on the distribution of chlorophyte species in the examined ponds (Fig. 1, Table 2). Among abiotic factors, NO_3,_ NH_4,_ TRP, conductivity and the pond size, were of the greatest importance (Table 2). In the case of biotic features, the abundance of filtrators had a significant influence. The abundance of the greatest group of chlorophyte taxa (e.g. *Ankistrodesmus falcatus, A. arcuatus, Pseudopediastrum boryanum*, *Pediastrum duplex*, *Stauridium tetras, Closterium tumidulum, Mucidosphaerium pulchellum*, *Scenedesmus arcuatus* var. *gracilis*, *Scemedesmus obtusus*) and the Shannon–Wiener Diversity Index were associated with the forest ponds and also with TRP (Fig. 1). The abundance of other chlorophytes, e.g. *Oocystis lacustris*, increased in field ponds. Another group of species (e.g. *Kirchneriella irregularis* var. *spiralis, Tetraedron minimum, Scenedesmus ecornis*) was found to positively correlate with the NH_4_ and conductivity. Species such as *Coelastrum astroideum* and *Monoraphidium contortum* were associated with the ponds of large size, and they also negatively correlated with NO_3_. However, nitrates positively influenced the abundance of *Tetradesmus lagerheimii*. A group of species with *Pandorina morum, Willea rectangularis, Desmodesmus armatus, Nephrochlamys willeana*, *Cosmarium trilobulatum* were negatively affected by filtrators, while the abundance of *Lemmermannia tetrapedia* and *Tetraedron triangulare* rose in the presence of the filtrating fraction of zooplankton.

**Table 2:** Results of CCA on relation between abundance of chlorophyte species and diversity and physical–chemical and biological parameters among field and forest ponds.

Variable	Lambda A	P	F
Catchment area (Field/Forest)	**0.18**	**0.002**	**3.79**
Nitrates (NO_3_)	**0.29**	**0.002**	**5.62**
Total reactive phosphorus (TRP)	**0.18**	**0.002**	**3.79**
Conductivity (Cond)	**0.19**	**0.002**	**3.98**
Size	**0.18**	**0.002**	**3.83**
Ammonium (NH_4_)	**0.13**	**0.002**	**3.02**
Filtrators	**0.20**	**0.002**	**4.09**
Depth	0.09	0.056	2.17
Nitrites (NO_2_)	0.13	0.068	3.00
pH	0.07	0.154	1.80
Rotifera	0.07	0.232	1.48

Values of p and F are calculated using Monte Carlo permutation test with 999 permutations. The overall percentage of explained variance was 25.95%. Bold = variables significantly adding to the model at p < 0.05 level (see Fig. 1).

There were no significant relationships between chlorophyte species and pond depth, NO_2_, pH and Rotifera (Table 2).

There were 44 chlorophytes taxa which occured exclusively in the field ponds (e.g. *Treubaria planctonica* and *Sorastrum spinulosum*) and 39 taxa in the forest ponds (e.g. *Kirchneriella cornuta, Cosmarium humile a*nd *Staurastrum alternans*) (Appendix S1).

### Relationships between chlorophyte species and environmental variables in different habitats (macrophyte and open water zones) of ponds

The open water zone was characterised by significantly higher values of pH and NH_4_, while pond size, DIN concentration and abundance of filtrators were lower in this water zone compared to the macrophyte-dominated stations (Table 3). The mean values of chlorophyte abundance, TRP and rotifera densities were higher in the open water stations, even though the differences were not significant. Other environmental variables did not vary between these two habitat types.

**Table 3:** Limnological parameters (SDV—Secchi disc visibility), number of chlorophytes taxa (N taxa) and individuals (No. ind.), phytoplankton diversity (Shannon—Shannon–Wiener Diversity Index) and zooplankton (Rotifera, Filtrators) abundance of different pond habitats (open water vs. macrophyte-dominated zone).

Type of habitat		Open water				Macrophytes				Mann–Whitney test	
Parameter	Unit	n samples	$\overline{x}$	Range	SD	n samples	$\overline{x}$	Range	SD	Z	p
Size	ha	64	0	0.002–4	1	64	1	0.0125–4	1	− 2.45	< 0.05
Depth	m	64	1	0.1–7	1	64	1	0.3–5	1	–	–
Temp	°C	64	23	14.1–35	4	64	23	10–31	4	–	–
pH		63	8	6.38–11	1	64	8	6.3–10	1	− 2.04	< 0.05
Cond	μS cm^−1^	64	756	26–2078	457	64	831	108.7–2078	428	–	–
SDV	m	64	1	0.02–4	1	64	1	0.1–4	1	–	–
O_2_	%	64	85	5–259	54	64	93	3–244	45	–	–
TRP	μg P l^−1^	64	258	0.0088–2181	475	64	175	0.0172–1323	324	–	–
DIN	mg l^−1^	64	2	0.31–9	2	64	4	0.253–160	20	3.27	< 0.001
NH_4_	mg l^−1^	64	1	0.02567–6	1	64	1	0–5	1	2.18	< 0.05
NO_3_	mg l^−1^	64	1	0–8	1	64	1	0.053–3	0	–	–
NO_2_	mg l^−1^	64	0	0–1	0	64	0	0	0	–	–
N taxa		64	18	1–49	11	64	22	5–53	12	–	–
No. ind	ind ml^−1^	64	3,967,025	0.01–156,400,005	19,835,029	64	2,057,530	0.006–38,873,445	5,590,647	–	–
Shannon		64	1	0–3	1	64	2	0.2089–3	1	–	–
Rotifera	ind l^−1^	64	4319	5.66667–42,795	9066	64	3081	7.5–27,921	4870	–	–
Filtrators	ind l^−1^	63	135	0–1988	316	64	664	0–4063	1020	− 4.83	< 0.001

The results of the Mann–Whitney test are given.

Generally, slightly more chlorophyte taxa were found on average among macrophytes than in the open water. The taxonomic diversity (Shannon) was also higher in the vegetated area compared to the open water zone.

The type of habitat (open water and macrophyte-dominated zones) was the significant determinant of chlorophyte community structure (Fig. 2, Table 4). Moreover, some physical–chemical parameters of water (NO_3_, NH_4_, TRP, conductivity), morphometric features (pond size and depth) and zooplankton (filtrators and Rotifera) were also of great importance (Table 4). A large group of chlorophyte taxa (e.g. *Mougeotia* sp., *Stauridium tetras*, *Scenedesmus obtusus*, *Monoraphidium contortum*, *Mucidosphaerium pulchellum*) was found to prefer macrophyte-dominated stations. At the same time, they were negatively correlated with the open water zone and conductivity. Another large group of chlorophytes (e.g., *Scenedesmus arcuatus* var. *gracilis*, *Pediastrum duplex*, *Closterium moniliferum*, *Closterium tumidulum*, *Cosmarium trilobulatum*, *Willea rectangularis*) was associated with higher values of NH_4_. Species such as *Monoraphidium tortile*, *Scenedesmus ecornis*, *Tetradesmus lagerheimii* and *Tetraedron minimum* were positively affected by total reactive phosphorus (TRP) and negatively by filtrator occurrence. One species (*Ankistrodesmus falcatus*) was positively affected by NO_3_. A group of species (e.g. *Cosmarium margaritatum, Lemmermannia tetrapedia, Tetraedron triangulare*) negatively correlated with this parameter, but positively with Rotifera and also with pond size and depth. Taking into consideration the impact of conductivity and the open water zone, a positive effect was found in the increasing abundance of e.g. *Pandorina morum*, *Nephrochlamys willeana*, *Desmodesmus armatus* and *Desmodesmus communis*. The other variables included (Table 4) had no significant effect on the distribution of chlorophyte species.

**Table 4:** Results of CCA on relation between abundance of chlorophyte species and diversity and physical–chemical and biological parameters among different habitats of ponds (Water vs. Macropytes).

Variable	Lambda A	P	F
Nitrates (NO_3_)	**0.29**	**0.002**	**5.58**
Total reactive phosphorus (TRP)	**0.20**	**0.002**	**4.09**
Size	**0.15**	**0.002**	**3.12**
Ammonium (NH_4_)	**0.18**	**0.004**	**3.91**
Habitat (Water/Macrophytes)	**0.14**	**0.004**	**2.87**
Conductivity (Cond)	**0.13**	**0.006**	**2.94**
Filtrators	**0.21**	**0.008**	**4.15**
Depth	**0.10**	**0.046**	**2.13**
Rotifera	**0.11**	**0.048**	**2.51**
Nitrites (NO_2_)	0.09	0.108	2.16
pH	0.08	0.138	1.76

Values of p and F are calculated using Monte Carlo permutation test with 999 permutations. The overall percentage of explained variance was 25.15%. Bold = variables significantly adding to the model at p < 0.05 level (see Fig. 2).

There were 37 species that occurred exclusively in the open water zones and 29 taxa in the macrophyte-dominated stations (Appendix S1).

### Chlorophyte functional groups: response to environmental variables in small water bodies

Chlorophyte taxa found in ponds have been classified into 14 phytoplankton functional groups, which were analysed in terms of type of the catchment and habitat (Appendix S1). Most of the identified green algae taxa belonged to the codon J (65 taxa), N (65 taxa), F (40 taxa), and X1 (28 taxa) according to the Reynolds Functional Groups (RFG) classification. The remaining groups were represented by a considerably lower number of taxa (1–9). The highest abundance was found in groups such as W_0_, F, J, X1 and X3.

The abundance of the codon TD, was significantly higher in the forest ponds, compared with the field ponds (Table 5). Furthermore, the abundances of the codons N and T were higher on average, however insignificantly, in the forest ponds, while those of the codons G, J, W_0_ and X_3_ in the field ponds.

**Table 5:** Abundance of each chlorophyte functional group (ind l^−1^) in different pond types (field vs. forest).

Type of pond	Field				Forest				Mann–Whitney test	
parameter	n samples	$\overline{x }$	Range	SD	n samples	$\overline{x }$	Range	SD	Z	P
F	81	520,477	0–8,374,665	1,379,288	47	493,814	0–10,043,000	2,006,881	**–**	**–**
G	81	20,844	0–483,000	81,395	47	13,304	0–560,000	81,650	**–**	**–**
J	81	727,427	0–13,360,000	1,810,540	47	379,970	0–3,406,000	703,170	**–**	**–**
K	81	988	0–80,000	8889	47	766	0–36,000	5251	**–**	**–**
MP	81	0	0	0	47	142	0–4000	694	**–**	**–**
N	81	27,360	0–429,000	70,522	47	52,707	0–1,152,000	174,941	**–**	**–**
T	81	5371	0–325,000	36,840	47	24,772	0–511,000	104,276	**–**	**–**
TD	81	7476	0–496,000	55,547	47	24,050	0–403,000	76,771	− 2.301	< 0.05
W1	81	321	0–24,000	2673	47	0	0	0	**–**	**–**
W_0_	81	1,942,481	0–156,160,000	17,349,606	47	31,099	1,056,000	159,399	**–**	**–**
X1	81	106,970	0–1,696,000	290,951	47	163,035	0–3,627,000	541,736	**–**	**–**
X2	81	7765	0–288,000	34,398	47	5681	0–80,000	15,968	**–**	**–**
X3	81	610,675	0–26,240,617	3,869,303	47	158,340	0–7,410,000	1,080,768	**–**	**–**

The results of Mann–Whitney test are given.

The codon N significantly prevailed in the open water zone, while the abundances of the codons T and TD were significantly higher in the macrophyte-dominated stations (Table 6). The codons G, K and W_0_, were higher in the open water zone, while the codons W_1_ and X_2_, had higher abundances in the macrophytes.

**Table 6:** Abundance of each chlorophyte functional group (ind l^−1^) in different pond habitats (open water vs. macrophyte-dominated zone).

Type of habitat	Open water				Macrophytes				Mann–Whitney test	
parameter	n samples	$\overline{x }$	Range	SD	n samples	$\overline{x }$	Range	SD	Z	P
F	64	396,273	0–10,043,000	1,382,319	64	625,101	0–9,732,000	1,849,186	–	–
G	64	31,844	0–560,000	112,415	64	4306	0–119,000	16,883	–	–
J	64	452,900	0–4,844,000	977,062	64	746,791	0.001–13,360,000	1,892,646	–	–
K	64	1250	0–80,000	10,000	64	563	0–36,000	4500	––	–
MP	64	0	0	0	64	104	0–4000	597	–	–
N	64	37,843	0–1,152,000	157,723	64	35,491	0–351,000	63,917	− 2.27	< 0.05
T	64	242	0–15,000	1875	64	24,747	0–511,000	97,632	− 2.39	< 0.05
TD	64	8307	0–403,000	51,852	64	18,817	0–496,000	74,876	− 2.27	< 0.05
W1	64	31	0–2000	250	64	375	0–24,000	3000	–	–
W_0_	64	2,455,687	0–156,160,000	19,518,148	64	25,604	0–1,056,000	138,362	–	–
X1	64	99,459	0–1,696,000	260,353	64	155,653	0–3,627,000	503,863	–	–
X2	64	4031	0–70,000	13,945	64	9969	0–288,000	38,449	–	–
X3	64	479,157	0–23,224,071	3,033,079	64	410,010	0–26,240,617	3,280,077	–	–

The results of Mann–Whitney test are given.

**Figure 3:**
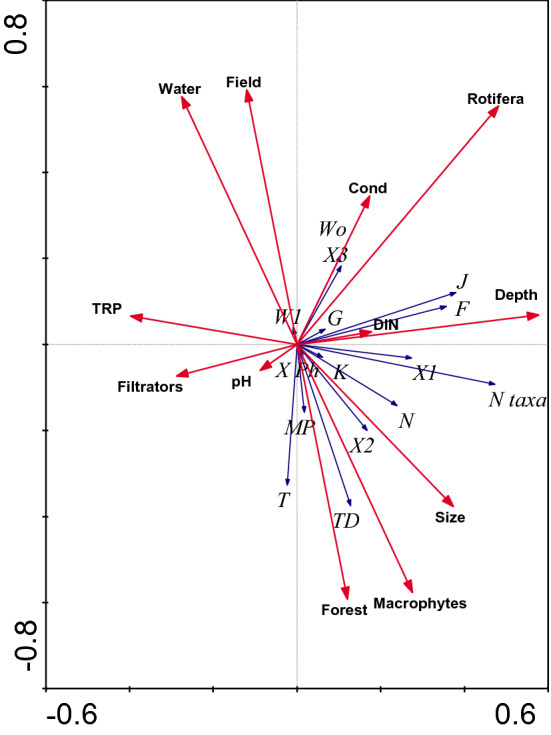
RDA diagram of the distribution of chlorophyte functional groups and number of taxa in relation to environmental factors in different types of catchment area (field and forest) and habitats (open water and macrophyte-dominated zones) of ponds.

Analysing the influence of the environmental variables on each chlorophyte codon (RDA analysis), the type of catchment area, the type of habitat as well as Rotifera abundance and the pond depth were found as significantly important (Fig. 3, Table 7). The codons T, TD and MP were associated with forest catchment and macrophytes. Moreover, codon X_2_ was associated only with the macrophyte-dominated stations. All these codons negatively correlated with the open water zone. Another group consisting of codons W_0_, X_3_ and G positively correlated with the abundance of Rotifera, while codons J, F, X_1_ and the number of chlorophyte taxa were positively affected by pond depth. There were no significant relationships between other variables and phytoplankton functional groups (Table 7).

**Table 7:** Results of RDA on relation between abundance of chlorophyte functional gropus and number of taxa and physical–chemical and biological parameters among different habitats of field and forest ponds.

Variable	Lambda A	P	F
Rotifera	**0.03**	**0.002**	**3.58**
Depth	**0.02**	**0.010**	**3.05**
Habitat (Water/Macrophytes)	**0.02**	**0.020**	**2.63**
Catchment area (Field/Forest)	**0.02**	**0.026**	**2.50**
Filtrators	0.01	0.064	1.91
Total reactive phosphorus (TRP)	0.01	0.144	1.60
Size	0.01	0.242	1.27
Conductivity (Cond)	0.01	0.268	1.20
pH	0.01	0.264	1.26
Dissolved inorganic nitrogen (DIN)	0.01	0.614	0.73

Values of p and F are calculated using Monte Carlo permutation test with 999 permutations. The overall percentage of explained variance was 14.7%. Bold = variables significantly adding to the model at p < 0.05 level (see Fig. 3).

## Discussion

### Response of chlorophytes to environmental variables in field vs. forest ponds

Our study demonstrated that human-originated transformation in the catchment area surrounding a small water body may influence the water conditions in terms of physical, chemical, and biological parameters as well as the ecological state of the aquatic environment in respect to green algae communities.

Chlorophytes inhabiting field ponds were more abundant compared with the forest ponds. This shows that field ponds, due to the higher values of TRP and water conductivity, created favorable conditions for chlorophyte development. The high concentrations of TRP and conductivity in aquatic environments are characteristic in the case of agricultural catchments exposed to anthropogenic pressure because of the inflow from the surrounding fertilized fields^42^. In this type of pond, we also observed significantly higher water temperatures and pH due to the lack of trees around them compared to the forest ponds, two factors which also positively influenced the growth of chlorophytes. Both the higher light intensity and the smaller size of the field ponds cause earlier warming up than the forest ponds and give an advantage to high light tolerant species. Moreover, it is well known that an increase in temperature stimulates the release of phosphorus from the bottom sediments, so this could be another reason for the higher levels of TRP in the field ponds. Our CCA analysis showed that TRP and conductivity were the strongest determinants of the distribution of chlorophyte species in the examined water bodies. We found a large group of dominant species indicated high values of TRP (e.g. *Ankistrodesmus falcatus, A. arcuatus, Monoraphidium griffithii, Pseudopediastrum boryanum*, *Pediastrum duplex, Scenedesmus obtusus, Scenedesmus arcuatus* var. *gracilis, Desmodesmus communis*, *Coelastrum microporum*), and another group of species (e.g. *Kirchneriella irregularis* var. *spiralis, Tetraedron minimum, Scenedesmus ecornis*) that preferred high levels of conductivity.

In the field ponds generally higher mean abundances of filtrators and Rotifera were observed. This could be another important factor stimulating the growth of chlorophytes and increasing their abundances by the resupply of nutrients through excretion^43,44^. On the other hand, the high densities of algae could be the factor that caused better zooplankton development, and therefore its abundance in field ponds was greater. Filtrating cladocerans and Rotifera also had a significant influence on the distribution of chlorophyte dominating species. However, even though the total abundance of both chlorophytes and filtering zooplankton was greater in the field ponds, CCA analysis revealed a negative relationship existing between filtrators and most dominant species of chlorophytes (e.g. *Pandorina morum, Willea rectangularis, Desmodesmus armatus, Nephrochlamys willeana, Cosmarium trilobulatum*). Only two chlorophyte species—*Lemmermannnia tetrapedia* and *Tetraedron triangulare*—co-occurred with cladoceran zooplankton. These latter species are very small compared to the species above and can therefore be overlooked by filtrators, which have a choice of larger and perhaps more nutritiously satisfying algae of the genus *Pandorina, Crucigeniella, Cosmarium* or *Nephrochlamys*, but still of a size suitable for zooplankton. It can also be interpreted in such a way that *Crucigenia* and *Tetraedron* are among the r-strategists that reproduce very quickly, so grazing pressure by zooplankton can stimulate their rapid development^45^ and thus they remain at a stable level.

Specific environmental conditions prevailing in the field ponds resulted in a high number of exclusive taxa^44^, found only in this type of water body. Moreover, a greater diversity of the representatives of different functional groups were found here, compared to the forest ponds.

Analyzing the distribution of chlorophytes in terms of phytoplankton functional groups^39,40^, we found that group W_1_ was represented by only one species, *Gonium pectorale.* This was especially noted in the field water bodies. This group is known to prefer small water bodies rich in organic matter from husbandry or sewage^40^, which suggests that the field catchment in our study migh be a supplier of these substances. It also proves that field surroundings are far more human impacted. In the field ponds we observed a higher abundance of chlorophytes belonging to the groups G (*Eudorina elegans*, *Pandorina morum*, *Pandorina smithii* and *Volvox aureus*), J (e.g. representatives of the genus *Actinastrum, Chlorotetraedron, Coelastrum, Crucigenia, Desmodesmus/Scenedesmus, Golenkinia, Pediastrum, Tetraedron, Tetrastrum, Westella, Willea/Crucigeniella)*, W_0_ (genera *Chlamydomonas, Chlorangiopsis, Chlamydomonadopsis, Planktococcomyxa/Coccomyxa*) and X_3_ (*Chlorella* sp.), typical for shallow nutrient-rich waters (G and J), ponds with extremely high organic contents (W_0_), and for shallow well-mixed layers (X_3_), according to classification given by Padisak et al.^40^. Considering that nitrogen compounds had a similar level in both types of ponds it can be stated that the representatives of the above mentioned functional groups of chlorophytes associated with the field ponds were presumably dependent on higher concentrations of TRP and conductivity and not that much on nitrogen concentrations.

In the forest ponds significantly higher values of water saturation were recorded compared to the field ponds. Moreover, the lack of inflow of fertilizers from the catchment area resulted in lower TRP concentrations, which along with lower water temperatures, pH and conductivity in the forest ponds may have contributed to the reduced abundance of chlorophytes compared to the field water bodies. RDA analysis showed that some dominant chlorophyte species (e.g. *Closterium moniliferum, Closterium tumidulum, Cosmarium trilobulatum* and *Mougeotia* sp.) were associated with this type of small water body. At the same time the abundance of these species was smaller in the field ponds. We also found that chlorophyte diversity (Shannon–Weaver index) was greater in the forest ponds. This suggests that water bodies located within the forested area, usually more natural ponds being less exposed to anthropogenic pressure, are characterized by greater biodiversity. Moreover, in this type of water body we found many exclusive species^39^, not reported from the field ponds. Interestingly, about the half of these taxa belonged to desmids, which prefer lower pH and conductivity^46^, conditions typical for forest ponds. This could be also a reason for the dominance of desmid species with the highest abundance/frequency, associated with forest ponds.

Taking into consideration the phytoplankton functional groups^39,40^ our study showed that the chlorophytes associated with forest ponds prefer mesotrophic waters (from the group TD: *Cladophora glomerata, Geminella turfosa, Geminella planctonica, Microspora* sp*., Netrium digitus, Oedogonium* sp., *Oocystidium ovale, Spirogyra* sp. *Zygnema* sp. and those belonging to the group N: mainly genera *Closterium, Cosmarium, Euastrum, Micrasterias, Staurastrum, Staurodesmus, Xanthidium*). This explains their greater share in the less fertile forest ponds. Another group associated with the forest ponds – T (*Mougeotia* sp., *Binuclearia lauterbornii)* contains species tolerant to light deficiency, so they were able to develop well in the more shaded water bodies located in the forest catchment.

### Chlorophyte community structure in two types of habitats (open water vs. macrophyte-dominated zone)

In our study, the type of habitat (open water and macrophyte-dominated zones) also had a significant structuring effect on chlorophytes. There were a group of species linked to the open water zone (*Pandorina morum, Nephrochlamys willeana, Oocystis lacustris, Scenedesmus armatus, Scenedesmus intermedius and Desmodesmus communis*), being negatively related to vegetated stations at the same time. Generally, we found here a higher mean abundance of chlorophytes compared to the macrophyte-dominated zones, possibly due to the higher values of nutrients such as NH_4_ and TRP, the conditions favouring the development of many algae species. The results of the CCA analysis with habitats confirmed the high importance of both nutritional factors in structuring the distribution of chlorophyte species. There was a group of species associated with a rise in the concentration of ammonium (e.g. *Scenedesmus arcuatus* var. *gracilis*, *Pediastrum duplex*, *Closterium moniliferum*, *Closterium tumidulum*, *Cosmarium trilobulatum*, *Willea rectangularis*) as well as with phosphates (*Monoraphidium tortile*, *Scenedesmus ecornis*, *Tetradesmus lagerheimii* and *Tetraedron minimum*). Generally, high abundance of chlorophytes in the open water area was accompanied by a small-sized fraction of zooplankton–rotifers. Therefore, rotifers had a lower impact on the distribution of chlorophytes than filtrators. The increasing numbers of cladocerans contributed to the lowering abundance of some chlorophytes, such as *Monoraphidium tortile, Scenedesmus ecornis, Tetradesmus lagerheimii* or *Tetraedron minimum*. This shows that filtrators, whose densities were significantly higher among macrophytes, were able to control the development of some chlorophyte species much more efficiently than small-bodied rotifers.

The effect of habitat was also visible in the case of phytoplankton functional groups^39,40^. We found that representatives of the group N (e.g. *Closterium, Cosmarium, Euastrum, Micrasterias, Staurastrum*) had a significantly higher mean abundance in the open water zones compared to the macrophyte-dominated zones. Interestingly, according to Padisak et al.^40^ group N prefers less fertile (mesotrophic) conditions, which is inconsistent with our results. However, we think that their association with the open water sites could be connected rather with the place/level where they live in the water column, rather than with the trophic state of water. The above mentioned chlorophytes taxonomically belong to desmids, which are mostly benthic organisms. Their greater quantitative share in the samples from the open water areas could be an effect of the intensive water mixing in the shallow ponds due to the lack of macrophytes. Neustupa et al.^47^ confirm that desmids are able to form tychoplanktonic communities due to water movements. In the samples collected from the macrophyte-dominated stations the mean abundance of desmids was generally lower, probably because of the macrophyte stabilizing effect. Aquatic plants are known to reduce turbidity and stabilize bottom sediments^48^, so they can prevent any intensive water mixing in ponds. In the examined open water stations, we also found a higher mean abundance of chlorophytes typical for shallow nutrient-rich waters (group G: *Eudorina, Pandorina, Volvox* and group K: *Radiococcus*) and/or for ponds with extremely high organic contents (group W_0_: e.g. *Chlamydomonas*), which proves that the sites lacking macrophytes were more fertile. Additionally, clearly more representatives from the codon J and X_1_ (typical for waters with high trophic levels) and a greater diversity of the representatives of different functional groups were recorded in the open water area compared to the macrophyte-dominated zones.

The macrophyte-dominated stations had more abundant communities of filtrators, as aquatic plants are known to provide a profitable shelter for zooplankton^49^. Cladoceran predominance among macrophytes may have been a force reducing green algae numbers. The chlorophytes of the investigated ponds were mostly small- or medium-size species. Their size distribution makes them a high quality food for zooplankton, particularly for cladoceran filtrators. According to RDA analysis apart from pond size, the presence of filtrators significanly reduced the abundance of several chlorophyte dominating species. The lower algae abundance among macrophytes compared to the open water zone could also be explained by competition between algae and macrophytes for light and nutrients^37,50^ and/or with the secretion of allelopathic substances e.g. by *Ceratophyllum demersum*^51^ inhibiting algal development. Our studies demonstrated that among chemical factors which clearly differentiated the two types of analysed habitat, TRP and NH_4_ significantly influenced the distribution of chlorophyte dominating species. The lower levels of these parameters in macrophyte-dominated zones suggest that the nutrient uptake by aquatic plants in the investigated water bodies was high. There are many reports on the decrease of nutrient concentrations by macrophytes^30,37,52^, which are consistent with our observations. Despite lower, compared to the open water zone, chlorophyte densities within the macrophyte-dominated zones there was a group of species (e.g. *Mougeotia* sp., *Pediastrum tetras*, *Scenedesmus obtusus*, *Monoraphidium contortum*) that selectively chose vegetated stands. Furthermore, we found a great number^29^ of exclusive chlorophyte species for macrophyte-dominated zones. Half of these taxa belong to desmids, which are often periphytic organisms associated with aquatic macrophytes^53,54^.

Preference towards macrophyte-dominated stations was also documented for two phytoplankton functional groups (T: *Mougeotia* sp. and *Binuclearia lauterbornii* and TD: e.g., *Spirogyra* sp., *Zygnema* sp., *Cladophora glomerata*, *Oedogonium* sp.) and one group which occurred exlusively among vegetated sites (MP—*Ulothrix*). Interestingly, all the representatives of these groups had a similar filamentous morphological form, which suggests that many of them are of epithytic origin, coexisting within aquatic plants. Two more groups—X_2_ (*Pseudodidymocystis/Didymocystis*, *Pteromonas*) and W_1_ (*Gonium pectorale*) were clearly affected by the presence of macrophytes. According to Padisak et al.^40^, codons TD and X_2_ indicate mesoeutrophic conditions and their higher abundances in the macrophyte-dominated zones also proves that plants contribute to lowering the trophic levels in the examined ponds. On the other hand, the relatively high abundance of the representative of the group W_1_ in these habitats suggests that macrophytes could enrich ponds with organic matter during the process of their decomposition.

Concluding, our results prove that different types of catchment area (field and forest) as well as different types of habitats (open water zone and macrophyte-dominated zone) create distinct, specific conditions (dependent on some physical–chemical and biological variables) for the occurrence of chlorophytes in small water bodies. We conclude that cosmopolitan chlorophytes undoubtedly respond to the level of habitat heterogeneity, contributing to the ecological assessment of small water bodies. Chlorophytes in particularl react to the level of human transformation in the ponds’ vicinities. This is why we suggest using them for water quality evaluation in ponds. This interdisciplinary research significantly broadens the knowledge, not only about the response of chlorophytes to physical–chemical parameters of water, but also about the food preferences of zooplankton for which green algae are the basic food, and vice versa about the impact of zooplankton on microalgae communities. The analyses provide valuable information on chlorophytes-zooplankton interactions and also about the relationships between chlorophytes and macrophytes. Received data emphasize the high value of field ponds, underestimated habitats particularly vulnerable to destruction in the agricultural landscape. The research will help to better understand the functioning of poorly studied small water bodies, which will contribute to the preservation of their biodiversity and protection against degradation. They will also be useful in the management of small water bodies based on the specificity of chlorophyte occurrence in various habitats and catchment type ponds. Moreover, these results are important in a broader context, as the interactions between the studied organisms and the physico-chemical parameters of water in small bodies of water are to some extent universal, so the analyses will broaden the knowledge about the functioning of larger bodies of water.

## Methods

Our study was conducted on a group of 66 small water bodies, which were situated in the area of the Wielkopolska Lakeland (Western Poland) (Appendix S2a, S2b). The ponds under study differed in size but none of them were larger than 4 ha, having a surface area of between 0.002 and 4 ha. Their maximum depth ranged between 0.1 and 7 m. The categorization of types of small water bodies (forest and field) was made based on the dominant type of catchment area surrounding the pond. In the case of ponds, the size of the catchment area is generally the smallest compared with other types of aquatic ecosystems such as lakes or especially rivers^55^. Therefore, based on the type of the pond’s direct catchment two groups of ponds were distinguished: field (40 water bodies) and forest^26^. The slightly unequal number of field and forest ponds reflected the natural types of surroundings occurring in the central part of Europe, where agricultural landscape prevails.

From the 66 ponds taken into account, a total number of 128 habitats were analysed. 64 sites within the area of open water were taken into consideration. Altogether, 40 sites among elodeids (with the most frequent sites located within *Ceratophyllum demersum, C. submersum, Myriophyllum spicatum, M. verticillatum, Potamogeton* spp., *Chara* spp.) and 24 sites among helophytes (with the most frequent sites located within *Phragmites australis, Typha latifolia, T. angustifolia, Schoenoplectus lacustris*) were analysed.

As not only the open water area but also macrophyte-dominated sites were analysed, the collection of material was restricted to the summer season (June–July) only. In order to avoid the diurnal variability of abiotic parameters and plankton abundance^56^, the samples were taken around midday from each site in triplicate (total number of samples: n = 384; in tables the average values from three replicates are presented).

Microalgae and zooplankton samples were taken from each site, using a plexiglass core sampler (∅ 50 mm; length 1.5 m) from among macrophyte beds. In the open water area, samples were taken using a calibrated vessel. Subsamples (1–2 L) were taken from randomly selected sites within each habitat to make up a 10 L sample. Microalgae samples were fixed in Lugol solution, and then were sedimented in the laboratory and thickened to a volume of 5–10 ml. Zooplankton samples were preserved with 96% ethanol. Qualitative and quantitative analyses of microalgae and zooplankton were determined with a light microscope (magnification 200 ×, 400 × and 1000 ×). The number of algae individuals was counted over at least 160 fields of a Fuchs–Rosenthal chamber (height: 0.2 mm, area: 0.0625 mm^2^). Single cells and coenobia were treated as individual units. In the case of trichomes, the standard length of the individual was considered as 100 μm. In the event of species forming colonies, a cover area of 400 μm^2^ was classified as a unit. The diversity index H was calculated. It was expressed with the Shannon–Wiener Diversity Index formula. This index unites information on species variety as well as on the relative distribution of species abundance. Algae taxa names and concepts were given in accordance with classifications set forth in Algaebase^57^.

The zooplankton samples were concentrated using a 45 μm mesh net and then fixed with 4% formalin. More details concerning zooplankton analyses are described in previous papers^20,58^. Basic abiotic parameters such as water temperature, dissolved oxygen, pH and conductivity were measured in-situ using a Portable Multiparameter Meter Sension 156 Hach (Hach Co., USA) but for multivariate analyses only the last two parameters were taken into account. Chemical analyses were conducted in the laboratory in order to determine total reactive phosphorus (TRP) and nitrogen forms. The nitrogen and phosphorus concentration were analysed with spectrophotometric methods according to Polish Standard Analytical Methods^59^: for ammonium nitrogen (N-NH4)—the method with Nessler’s reagent, for nitrite nitrogen (N-NO2)—with sulphanilic acid; for nitrate nitrogen (N-NO3)—with sodium salicylate and for total reactive phosphorus (TRP)—with ascorbic acid. Details describing nutrient analyses have been also given in previous papers, e.g.^60^.

To better illustrate and understand the various survival strategies and habitat preferences of chlorophytes species, we divided them into groups (codons) according to the Reynolds Functional Groups (RFG) classification scheme^39^, updated and expanded by Padisak et al.,^40^. It is a widely used method in ecological studies of freshwater phytoplankton and clusters species with similar environmental sensitivities and tolerances but belong to different systematic groups. The chlorophyte taxa in our study belonged to the following RFG groups (codons) related to specific habitat preferences: F—clear, meso-eutrophic lakes, tolerant to high turbidity; G—nutrient-rich conditions in stagnating water columns, tolerant to high light; J—shallow enriched lakes, ponds and rivers; K—short nutrient-rich water columns; MP—inorganically turbid shallow lakes; N—mesotrophic epilimnia, tolerant to nutrient deficiency, mixed layer; T—deep well-mixed epilimnia, tolerant to light deficiency; TD—mesotrophic standing waters or rivers with emergent and submerged macrophytes; W_1_—small organic ponds (rich in organic matter from husbandry or sewage); W_0_—rivers and ponds with extremely high organic contents; X_1_—shallow mixed layers, eu-hypertrophic environments; X_2_—shallow mixed layers, meso-eutrophic environments; X_3_—shallow mixed layers, oligotrophic environments; X_Ph_—calcium, alkaline, small ponds.

The multivariate analyses, conducted with Canoco for Windows 4.5 software, were used to determine the distribution of chlorophyte dominating species or chlorophyte functional group abundance distribution in relation to environmental factors. The type of analysis (DCA—detrended correspondence analysis—as preselection for further RDA or CCA analysis, RDA—redundancy analysis and CCA—canonical correspondence analysis) was chosen with regard to the rules given by ter Braak and Šmilauer^61^ and Lepš and Šmilauer^62^. Species that were characterized by high frequency (≥ 20% of the samples) and high average abundance (> 10% of the abundance of chlorophyte community in a sample) were selected for the analyses.

The Mann–Whitney test (Statistica 10, StatSoft) was applied for the determination of the effect of habitat (the open water zone and macrophyte sites) and type of pond catchment area (field and forest) on environmental parameters, biotic features (filtrators and rotifers) as well as chlorophyte species and functional group occurrence.

### Ethics declarations

Experimental research and field studies on plants, including the collection of plant material, comply with relevant institutional, national, and international guidelines and legislation.

## Supplementary Information

Supplementary Information 1.

Supplementary Information 2.

Supplementary Information 3.

## Data Availability

The datasets used and/or analysed during the current study available from the corresponding author on reasonable request.
