# Supplementary material for: Chlorophytes response to habitat complexity and human disturbance in the catchment of small and shallow aquatic systems
Source: Sci Rep. 2022 Jul 29;12:13050. doi: 10.1038/s41598-022-17093-3 (PMC9338304; doi:10.1038/s41598-022-17093-3)
Supplement: Supplementary file 1 — Supplementary Information 1. [file 41598_2022_17093_MOESM1_ESM.pdf]

Appendix 1 – Frequency (%) of certain chlorophyte species in various pond types (field and forest) and habitats (macrophytes and open water) of the examined small water bodies. Functional Groups of phytoplankton (RFG) of each species is also given.

name of taxa (synonym)	coda	macro phytes	water	field	forest
Acanthosphaera zachariasii Lemm.	J	1	1	0	2
Actinastrum aciculare Playf.	J	3	3	3	3
Actinastrum hantzschii Lagerheim	J	5	2	3	4
Acutodesmus raciborskii (Woloszynska) Tsarenko & D.M.John (Scenedesmus raciborskii Wołosz.)	J	5	5	5	5
Ankistrodesmus arcuatus Korshikov (Monoraphidium arcuatum (Korš.) Hind.)	X1	13	9	12	9
Ankistrodesmus falcatus (Corda) Ralfs	X1	10	12	15	7
Ankistrodesmus stipitatus Komárková-Legnerová	X1	5	8	8	5
Ankyra ancora (G.M. Smith) Fott	X1	0	1	0	1
Ankyra sp. Fott	X1	0	1	0	1
Ankyra judayi (G.M.Smith) Fott	X1	2	4	3	3
Binuclearia lauterbornii (Schmidle) Proshkina-Lavrenko (Planktonema lauterbornii Skuja)	T	2	1	2	0
Botryococcus braunii Kütz.	F	9	5	9	5
Characium sp. A. Braun in Kütz.	X1	0	1	0	1
Chlamydomonadopsis B.Fott	Wo	1	2	2	1
Chlamydomonas bergii Nygaard	Wo	0	1	1	0
Chlamydomonas globosa Snow	Wo	1	1	1	1
Chlamydomonas passiva Skuja	Wo	2	2	2	3
Chlamydomonas reinhardtii Dangeard	Wo	3	2	2	3
Chlamydomonas sp. Ehrenb.	Wo	2	3	4	1
Chlorangiopsis sp.	Wo	0	2	1	1
Chlorella sp. Beijerinck	X3	1	2	2	2
Chlorotetraedron bitridens (Beck-Mann.) Kovač.	J	2	0	2	1
Chlorotetraedron incus (Teiling) Komárek & Kovácik (Tetraedron incus (Teiling) G.M.Smith)	J	0	2	1	1
Cladophora glomerata (L.) Kützing	TD	1	0	0	1
Closterium acerosum (Schrank) Ehrenb. ex Ralfs	N	2	2	3	0
Closterium aciculare T. West.	N	5	3	6	2
Closterium acutum Bréb.	N	5	8	10	3
Closterium acutum v. linea (Perty) W. & GaS. West.	N	2	1	2	1
Closterium acutum var. variabile (Lemm.) Krieg.	N	1	1	1	1
Closterium ceratium Perty	N	6	4	8	2
Closterium directum Archer	N	0	1	0	1
Closterium ehrenbergii Meneghini ex Ralfs	N	6	2	5	2
Closterium gracile Bréb. ex Ralfs	N	9	4	5	8
Closterium incurvum Bréb.	N	6	3	2	7
Closterium kuetzingii Breb.	N	1	1	1	1
Closterium pronum Breb	N	1	0	1	0
Closterium setaceum Ehrenberg ex Ralfs	N	0	1	0	1
Closterium sp.	N	2	4	6	0
Closterium strigosum Bréb.	N	4	2	3	3
Closterium tumidulum F.Gay	N	15	7	18	4
Closterium lunula Ehrenberg & Hemprich ex Ralfs	N	3	0	2	2
Closterium moniliferum Ehrenberg ex Ralfs	N	17	5	14	9

Coelastrum astroideum De Notaris	J	27	22	33	16
Coelastrum microporum Nägeli	J	24	19	27	16
Coelastrum proboscideum Bohlin	J	0	2	1	1
Coelastrum speciosum (Wolle) Brun.	J	1	3	2	2
Coenococcus planctonicus Korshikov (Eutetramorus planctonicus (Korshikov) Bourrelly)	F	0	1	1	0
Coenocystis planctonica Kors.	F	6	2	1	7
Comasiella arcuata var. platydisca (G.M.Smith) E.Hegewald & M.Wolf (Scenedesmus arcuatus v. platydiscus G. M. Smith)	J	5	2	5	2
Cosmarium bioculatum Bréb. ex Ralfs	N	0	1	0	1
Cosmarium botrytis Menegh ex Ralfs	N	6	2	8	1
Cosmarium cymatopleurum Nordstedt	N	0	1	0	1
Cosmarium dentiferum Corda ex Nordstedt	N	7	3	3	7
Cosmarium fontigenum Nordstedt	N	1	0	1	0
Cosmarium granatum Bréb. ex Ralfs	N	9	2	9	2
Cosmarium humile (Gay) Nordstedt in De Tony	N	5	0	0	5
Cosmarium impressulum Elfving	N	7	5	9	2
Cosmarium laeve Rabenhorst	N	9	9	12	7
Cosmarium lundellii Delponte	N	0	1	0	1
Cosmarium margaritatum (P.Lundell) J.Roy & Bisset	N	12	2	6	7
Cosmarium meneghinii Breb. ex Ralfs	N	1	0	1	0
Cosmarium neodepressum G.J.P.Ramos & C.W.N.Moura (Cosmarium depressum (Näg.) Lund)	N	1	0	1	0
Cosmarium phaseolus Bréb. in Ralfs	N	2	1	1	2
Cosmarium quadratulum (Gai) de Toni	N	1	0	1	0
Cosmarium rectangulare Grun.	N	2	1	1	2
Cosmarium regnellii Wille	N	2	2	2	2
Cosmarium reniforme (Ralfs) Archer	N	0	1	0	1
Cosmarium sexnotatum var. tristriatum (Lütkem.) Schmidle	N	1	0	0	1
Cosmarium sp. Corda ex Ralfs	N	3	8	5	5
Cosmarium succisum West	N	0	1	0	1
Cosmarium trilobulatum Reinsch	N	15	8	16	7
Cosmarium venustum (Breb.) Arch.	N	1	1	2	0
Cosmarium constrictum Delponte	N	4	0	1	3
Crucigenia fenestrata (Schmidle) Schmidle	J	2	2	2	2
Crucigenia quadrata Morren	J	9	7	10	5
Desmatractum indutum (Geitler) Pascher	J	1	2	1	2
Desmodesmus abundans (Kirchner) E.H.Hegewald (Scenedesmus quadrispina Chodat.)	J	6	7	13	1
Desmodesmus armatus (Chodat) E.H.Hegewald (Scenedesmus armatus Chodat)	J	13	16	17	12
Desmodesmus bicaudatus (Dedusenko) P.M.Tsarenko (Scenedesmus bicaudatus Dedusenko)	J	1	2	2	1
Desmodesmus bicellularis (Chodat) S.S.An, T.Friedl & E.Hegewald (Didymocystis bicellularis (Chod.) Kom.)	X1	0	1	0	1
Desmodesmus communis (Hegew.) Hegew.	J	34	26	34	26
Desmodesmus denticulatus (Lagerheim) S.S.An, T.Friedl & E.Hegewald (Scenedesmus denticulatus Lagerh.)	J	4	2	5	2

Desmodesmus intermedius (Chodat) E.Hegewald (Scenedesmus intermedius Chodat)	J	14	9	13	10
Desmodesmus intermedius var. acutispinus (Roll) E.Hegewald (Scenedesmus intermedius var. acutispinus (Y.V.Roll) E.Hegwald & An)	J	1	2	2	1
Desmodesmus maximus (West & G.S.West) Hegewald (Scenedesmus maximus (W. et. G.S. West) Chod.)	J	5	5	9	2
Desmodesmus opoliensis (P.G.Richter) E.Hegewald (Scenedesmus serratus (Corda) Bohlin)	J	9	13	18	4
Desmodesmus serratus (Corda) S.S.An, Friedl & E.Hegewald (Scenedesmus serratus (Corda) Bohlin)	J	1	0	1	0
Desmodesmus spinosus (Chodat) E.Hegewald (Scenedesmus spinosus Chodat.)	J	2	2	1	2
Dictyosphaerium chlorelloides (Naum.) Kom. et Perman	F	9	6	10	5
Dictyosphaerium ehrenbergianum Nägeli	F	5	3	5	4
Dictyosphaerium reniforme Bulnheim	F	1	0	1	0
Elakatothrix acuta Pascher	F	0	1	1	0
Elakatothrix gelatinosa Wille	F	1	2	1	2
Elakatothrix spirochroma (Reverdin) Hindák	F	9	8	11	5
Euastrum gemmatum Ralfs	N	0	1	0	1
Euastrum oblongum Ralfs	N	0	1	0	1
Euastrum pinnatum Ralfs	N	0	1	0	1
Euastrum sp. Ehrenb.	N	1	2	0	2
Eudorina elegans Ehr.	G	2	1	3	0
Fotterella tetrachlorelloides Buck	F	1	1	2	0
Geminella planctonica (Bolochonzew) G.L.Tiwari & D.C.Pandey, 1972 (Gloeotila planctonica Bolochonzev)	TD	0	1	1	0
Geminella turfosa (Skuja) Ramanathan, 1964(Gleotila turfosa Skuja)	TD	2	2	2	2
Golenkinia radiata Chodat	J	3	4	5	2
Gonatozygon aculeatum Hastings	N	1	0	1	0
Gonatozygon brebissonii De Bary	N	0	1	0	1
Gonium pectorale O. F. Müller	W1	2	2	3	1
Hariotina reticulata P.A.Dangeard (Coelastrum reticulatum (Dang.) Senn)	J	4	4	5	2
Heimansia pusilla (L.Hilse) Coesel	N	1	0	1	0
Hindakia tetrachotoma (Printz) C.Bock, Pröschold & Krienitz (Dictyosphaerium tetrachotomum Printz.)	F	0	2	1	1
Kirchneriella cornuta Korshikov	F	2	1	0	3
Kirchneriella irregularis v. spiralis Korš.	F	12	8	15	5
Kirchneriella lunaris (Kirch.) Mob.	F	1	1	2	0
Kirchneriella obesa (West) West & G.S.West	F	2	1	1	2
Koliella longiseta (Vischer) Hind.	F	2	2	2	3
Korschikoviella limnetica (Lemm.) Silva	X1	0	1	0	1
Lacunastrum gracillimum (West & G.S.West) H.A.McManus (Pediastrum duplex v. gracillimum W. et G.S. West)	J	1	2	2	1
Lagerheimia ciliata (Lagerh.) Chodat	X1	6	3	4	5
Lagerheimia citriformis (Snow) Collins	X1	4	2	5	1
Lagerheimia genevensis (Chod.) Chod.	X1	1	0	0	1
Lagerheimia longiseta (Lemmermann) Printz	X1	1	0	1	0

Lagerheimia marssonii Lemm.	X1	2	1	2	1
Lagerheimia wratislaviensis Schroeder	X1	0	1	1	0
Lemmermannia punctata (Schmidle) C.Bock & Krienitz (Tetrastrum punctatum (Schmidle) Ahlstr. et Tiff.)	J	2	3	4	1
Lemmermannia tetrapedia (Kirchner) Lemmermann (Crucigenia tetrapedia (Kirchner) W. et G.S. West)	J	13	13	17	9
Messastrum gracile (Reinsch) T.S.Garcia (Ankistrodesmus gracilis (Reinsch) Korshikov)	X1	3	5	2	5
Micractinium pusillum Fresenius	F	5	5	6	4
Micrasterias thomasiniana Archer	N	0	1	0	1
Microspora sp.	TD	3	1	0	4
Monactinus simplex (Meyen) Corda (Pediastrum simplex Meyen)	J	0	2	1	1
Monactinus simplex var. echinulatum (Wittrock) Pérez, Maidana & Comas (Pediastrum simplex var. echinulatum Wittrock)	J	1	1	0	2
Monoraphidium circinale (Nyg.) Nyg	X1	4	3	2	5
Monoraphidium contortum (Thur.) Kom.-Legn.	X1	20	16	20	16
Monoraphidium griffithii (Berk.) Kom.-Legn.	X1	18	23	24	16
Monoraphidium irregulare (G. M. Smith) Kom.-Legn.	X1	2	2	2	3
Monoraphidium komarkovae Nygaard	X1	9	8	9	7
Monoraphidium minutum (Nag.) Kom.-Legn.	X1	2	2	3	1
Monoraphidium mirabile (W. et G. S. West) Pankow	X1	0	1	0	1
Monoraphidium pusillum (Printz) Kom.-Legn.	X1	1	1	0	2
Monoraphidium tortile (W. et G. S. West) Kom.-Legn.	X1	7	10	10	7
Mougeotia sp.	T	20	9	18	10
Mucidosphaerium pulchellum (H.C.Wood) C.Bock, Proschold & Krienitz (Dictyosphaerium pulchellum Wood)	F	9	13	13	10
Mychonastes jurisii (Hindák) Krienitz, C.Bock, Dadheech & Proschold (Pseudodictyosphaerium jurisii Hindak)	F	2	2	2	1
Neglectella solitaria (Wittrock) Stenclová & Kastovsky (Oocystis solitaria Wittrock)	F	9	5	12	3
Neodesmus danubialis Hindák	J	1	1	1	1
Nephrochlamys willeana (Printz.) Korš.	F	15	20	23	12
Nephrocytium agardhianum Nägeli	F	5	2	5	2
Nephrocytium lunatum West	F	2	1	3	0
Netrium digitus (Ehrenb.) Itzigsohn et Rothe	TD	0	1	0	1
Oedogonium sp.	TD	2	1	2	2
Oocystidium ovale Korš.	TD	1	1	2	0
Oocystis borgei Snow	F	2	2	3	0
Oocystis lacustris Chodat	F	21	15	22	14
Oocystis marssonii Lemm.	F	2	0	1	1
Oocystis parva W. et G.S. West	F	9	8	11	5
Oocystis pelagica Lemm.	F	1	2	2	0
Oocystis rhomboidea Fott	F	2	2	4	0
Oocystis sp. A. Braun	F	1	1	1	1
Pandorina morum (O. F. Müller) Bory	G	13	7	13	6
Pandorina smithii Chodat	G	4	5	4	5
Parapediastrum biradiatum (Meyen) E.Hegewald (Pediastrum biradiatum Meyen)	J	5	2	3	4

Parapediastrum biradiatum var. longecornutum (Gutwinski) Tsarenko (Pediastrum biradiatum var. longecornutum Gutwinski)	J	0	1	1	0
Pectinodesmus regularis (Svirenko) E.Hegewald, M.Wolf, Al.Keller, Friedl & Krienitz (Scenedesmus regularis Swirenko)	J	5	8	11	2
Pediastrum duplex Meyen	J	19	16	20	15
Phacotus lenticularis (Ehr.) Stein	X Ph	1	0	1	0
Planctococcus sphaerocystiformis Korshikov	F	2	2	2	2
Planktococcomyxa lacustris (Chodat) Kostikov, Darienko, Lukesová & Hoffmann (Coccomyxa lacustris)	Wo	1	0	1	0
Pleurotaenium trabecula Nägeli	N	2	0	1	1
Pseudodidymocystis planctonica (Korshikov) E.Hegewald & Deason (Didymocystis planctonica Kors.)	X2	4	5	5	4
Pseudopediastrum boryanum var. longicorne (Reinsch) Tsarenko	J	5	3	4	4
Pseudopediastrum boryanum (Turpin) E.Hegewald (Pediastrum boryanum (Turp.) Menegh.)	J	26	24	31	19
Pseudopediastrum brevicorne (A.Braun) M.Jena & C.Bock (Pediastrum boryanum var. brevicorne A. Br.)	J	2	5	6	2
Pseudopediastrum cornutum (Raciborski) Lenarczyk (Pediastrum boryanum var. cornutum (Racib.) Sulek)	J	2	1	2	2
Pseudoquadrigula obtusa (Korshikov) Tsarenko (Kirchneriella obtusa (Kors.) Kom.)	F	0	1	0	1
Pteromonas aculeata Lemm.	X2	2	0	2	1
Pteromonas angulosa (Carter) Lemm.	X2	5	3	5	3
Pteromonas cordiformis Lemm.	X2	3	2	3	2
Radiococcus nimbatus (de Wildeman) Schmidle	K	2	1	2	0
Radiococcus planctonicus Lund.	K	1	0	0	1
Raphidocelis danubiana (Hindák) Marvan, Komárek & Comas (Kirchneriella contorta (Schmidle) Bohlin)	F	11	9	16	3
Raphidocelis danubiana var. elegans (Playfair) Taşkin & Alp (Kirchneriella contorta var. elegans (Playf.) kom.)	F	2	0	1	2
Raphidocelis microscopica (Nygaard) Marvan, Komárek & Comas (Kirchneriella microscopica Nygaard)	F	1	1	0	2
Raphidonema planctonicum (Hindák) Hoham	F	1	1	2	0
Scenedesmus arcuatus v. gracilis (Hortob.) Hind.	J	12	5	11	5
Scenedesmus breviaculeatus Chodat	J	0	2	0	2
Scenedesmus ecornis (Ehrenb.) Chodat	J	33	25	37	21
Scenedesmus ellipticus Corda (Scenedesmus linearis Kom.)	J	6	3	7	2
Scenedesmus naegelii Breb.	J	0	1	1	0
Scenedesmus obtusus Meyen	J	16	14	20	10
Scenedesmus subspicatus Chodat (Scenedesmus gutwinskii Chodat)	J	13	13	16	9
Schroederia planctonica (Skuja) Philipose	X1	2	5	5	2
Schroederia setigera (Schröder) Lemmermann	X1	1	2	2	0
Selenastrum bibraianum Reinsch (Ankistrodesmus bibraianus (Reinsch.) Kors.)	X1	5	9	9	5

Sorastrum spinulosum Nägeli	J	3	2	5	0
Sphaerocystis planctonica (Kors.) Bourr.	F	7	7	11	3
Sphaerocystis schroeteri Chodat	F	1	1	0	2
Spirogyra sp.	TD	16	5	10	11
Staurastrum alternans Breb.	N	2	0	0	2
Staurastrum gracile Ralfs	N	5	5	4	5
Staurastrum manfeldtii Delponte	N	1	0	1	0
Staurastrum margaritaceum Meneghini ex Ralfs	N	0	1	0	1
Staurastrum paradoxum Meyen ex Ralfs	N	2	1	2	1
Staurastrum polymorphum Brébisson	N	4	3	4	3
Staurastrum pseudotetracerum (Nordstedt) West & G.S.West	N	2	2	4	0
Staurastrum rugulosum Brébisson ex Ralfs	N	1	0	1	0
Staurastrum sp. Meyen	N	3	2	5	1
Staurastrum stauroton A.M.Scott & Prescott	N	1	0	0	1
Staurastrum tetracerum Ralfs ex Ralfs	N	7	5	6	6
Stauridium tetras (Ehrenberg) E.Hegewald (Pediastrum tetras (Ehrenb.) Ralfs)	J	24	17	23	19
Staurodesmus dejectus (Breb.) Teiling	N	1	1	1	1
Staurodesmus dejectus var. apiculatus (Brébisson) Croasdale (Staurodesmus apiculatus (Bréb.) Teiling)	N	2	2	2	2
Tetrachlorella alternans (G.M. Smitch) Kors.	J	3	2	5	1
Tetradesmus crocini Fott & Komárek	J	0	1	1	0
Tetradesmus lagerheimii M.J.Wynne & Guiry (Scenedesmus acuminatus (Lagerheim) Chodat)	J	10	11	14	7
Tetradesmus obliquus (Turpin) M.J.Wynne (Scenedesmus acutus Meyen; Scenedesmus obliquus (Turp.) Kütz.)	J	23	21	24	20
Tetraedron caudatum (Corda) Hansgirg	J	19	15	20	13
Tetraëdron minimum (A.Braun) Hansgirg	J	32	34	42	24
Tetraedron triangulare Kors.	J	16	16	21	11
Tetrastrum elegans Playfair	J	1	0	0	1
Tetrastrum glabrum (Roll) Ahlstrom & Tiffany	J	9	9	9	9
Tetrastrum staurogeniiforme (Schröder) Lemmermann	J	5	2	4	3
Treubaria planctonica (G.M.Smith) Korshikov	F	2	2	5	0
Treubaria triappendiculata C.Bernard	F	2	5	2	4
Ulothrix sp. 2 Kütz.	MP	0	1	1	0
Ulothrix sp.1 Kütz.	MP	2	1	1	2
Ulothrix zonata (F.Weber & Mohr) Kützing	MP	3	2	3	2
Verrucodesmus verrucosus (Y.V.Roll) E.Hegewald (Scenedesmus verrucosus Roll)	J	2	1	2	0
Volvox aureus Egrenb.	G	1	1	2	0
Westella botryoides (W. West) De Wild.	J	5	4	5	5
Willea apiculata (Lemmermann) D.M.John, M.J.Wynne & P.M.Tsarenko (Crucigeniella apiculata (Lemm.) Kom.,)	J	4	4	6	2
Willea neglecta (Fott & H.Ettl) D.M.John, M.J.Wynne & P.M.Tsarenko (Crucigeniella neglecta (Fott et Ettl) Kom.)	J	1	1	2	0
Willea rectangularis (A.Braun) D.M.John, M.J.Wynne & P.M.Tsarenko (Crucigeniella rectangularis (Näg.) Komárek)	J	13	12	16	8

Xanthidium brebissonii Ralfs	N	0	1	0	1
Zygnema sp.	TD	4	1	1	4
