# Supplementary figures and images for: Chlorophytes response to habitat complexity and human disturbance in the catchment of small and shallow aquatic systems
Source: Sci Rep. 2022 Jul 29;12:13050. doi: 10.1038/s41598-022-17093-3 (PMC9338304; doi:10.1038/s41598-022-17093-3)

Wielkopolska region

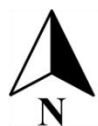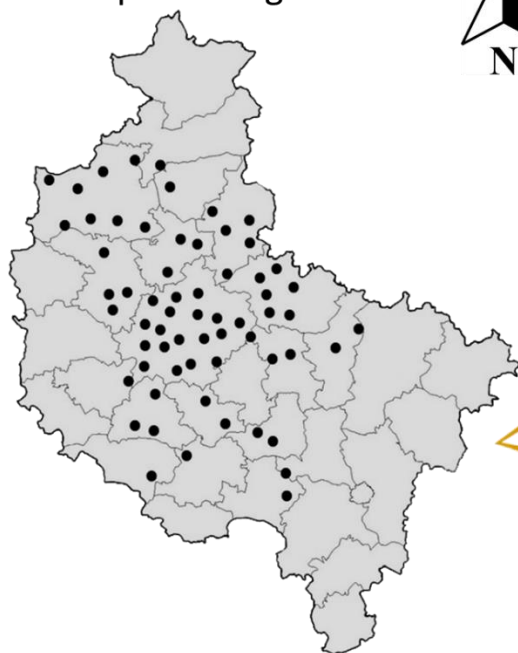

POLAND

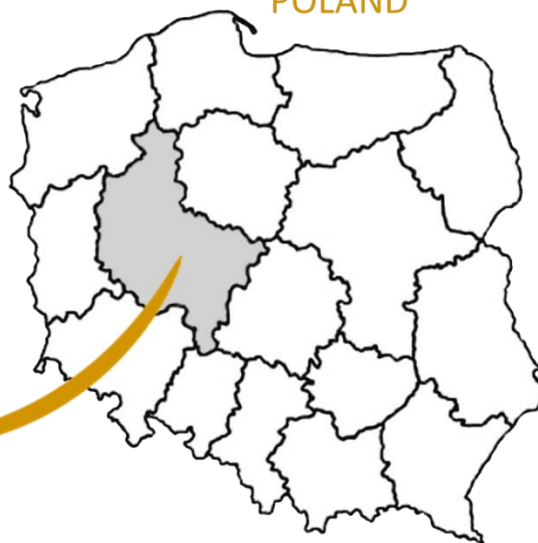

Supplement: Supplementary file 2 — Supplementary Information 2. [file 41598_2022_17093_MOESM2_ESM.pdf]
