# Supplementary material for: Chlorophytes response to habitat complexity and human disturbance in the catchment of small and shallow aquatic systems
Source: Sci Rep. 2022 Jul 29;12:13050. doi: 10.1038/s41598-022-17093-3 (PMC9338304; doi:10.1038/s41598-022-17093-3)
Supplement: Supplementary file 3 — Supplementary Information 3. [file 41598_2022_17093_MOESM3_ESM.pdf]

Appendix 2b - Position of studied water bodies

pond number	coordinates		pond types
1	52°12'27.03" N	16°59'19.05" E	Field
2	52°25'23.41" N	16°45'00.22" E	Field
3	52°22'03.16" N	16°44'12.17" E	Field
4	52°23'19.49" N	16°55'45.75" E	Field
5	52°22'23.03" N	16°51'28.94" E	Field
6	52°22'19.19" N	16°51'38.38" E	Field
7	52°26'30.97"N	16°57'32.48" E	Field
8	52°28'27.79" N	16°37'51.62" E	Field
9	52°28'41.17" N	16°38'03.47" E	Field
10	52°25'11.43" N	16°46'31.99" E	Field
11	52°46'02.39" N	16°29'37.92" E	Field
12	52°27'51.59" N	16°45'53.71" E	Field
13	52°40'37.51" N	17°38'50.79" E	Field
14	52°40'24.45" N	17°38'39.92" E	Field
15	52°40'22.31" N	17°38'34.38" E	Field
16	52°40'00.88" N	17°30'19.67" E	Field
17	52°37'31.35" N	17°36'35.97" E	Field
18	52°36'16.65" N	17°34'55.43" E	Field
19	52°40'56.49" N	17°38'25.83" E	Field
20	52°37'14.46" N	17°33'10.28" E	Field
21	51°58'48.48" N	16°41'01.04" E	Field
22	51°55'33.78" N	16°37'00.15" E	Field
23	52°22'03.16" N	16°44'12.17" E	Field
24	51°57'51.12" N	16°44'00.19" E	Field
25	51°57'50.02" N	16°43'54.02" E	Forest
26	52°00'06.01" N	16°58'32.87" E	Forest
27	52°02'32.48" N	16°52'10.42" E	Forest
28	52°01'44.05" N	16°57'23.14" E	Forest
29	52°25'23.41" N	16°45'00.22" E	Field
30	52°12'26.06" N	16°57'47.33" E	Field
31	52°12'31.01" N	16°58'24.03" E	Forest
32	52°11'35.07" N	16°59'11.07" E	Forest
33	52°10'35.01" N	16°59'10.02" E	Forest
34	52°10'31.54" N	16°56'16.07" E	Forest
35	52°10'08.04" N	16°57'27.05" E	Forest
36	52°11'59.77" N	16°58'53.02" E	Forest
37	52°15'18.46" N	16°49'51.16" E	Forest
38	52°46'59.72" N	16°27'39.93" E	Forest
39	52°46'54.42" N	16°26'51.92" E	Forest
40	52°41'29.21" N	16°33'50.86" E	Field
41	52°28'08.59" N	16°58'55.02" E	Forest
42	52°51'58.23" N	16°26'43.02" E	Forest
43	52°51'43.86" N	16°25'43.62" E	Forest
44	52°46'16.23" N	16°28'35.14" E	Forest
45	52°42'19.15" N	16°29'11.34" E	Field
46	52°42'42.69" N	16°30'36.72" E	Forest
47	52°47'01.87" N	16°21'21.73" E	Forest
48	52°47'10.18" N	16°25'40.96" E	Forest
49	52°24'23.43" N	16°50'39.39" E	Forest
50	52°29'15.32" N	16°56'11.51" E	Forest
51	52°28'03.09" N	16°55'59.97" E	Forest
52	52°23'38.61" N	16°49'30.51" E	Forest
53	52°28'08.59" N	16°58'55.02" E	Forest
54	52°39'02.49" N	17°37'57.25" E	Forest
55	52°03'33.54" N	16°49'20.19" E	Field
56	52°29'02.01"N	16°37'08.20"E	Field
57	52°28'47.80" N	16°37'08.90"E	Field
58	52°29'24.00"N	16°36'06.10"E	Field
59	52°28'52.60"N	16°36'06.10"E	Field
60	52°12'26.06" N	16°57'47.33" E	Field
61	52°41'29.21" N	16°33'50.86" E	Field
62	52°03'33.54" N	16°49'20.19" E	Field
63	52°26'30.97"N	16°57'32.48" E	Field
64	52°40'56.49" N	17°38'25.83" E	Field
65	52°22'23.03" N	16°51'28.94" E	Field
66	52°29'24.00"N	16°36'06.10"E	Field
